# Characterization of the iPhone LiDAR-Based Sensing System for Vibration Measurement and Modal Analysis

**DOI:** 10.3390/s23187832

**Published:** 2023-09-12

**Authors:** Gledson Rodrigo Tondo, Charles Riley, Guido Morgenthal

**Affiliations:** 1 Chair of Modelling and Simulation of Structures, Bauhaus University Weimar, Marienstr. 13, 99423 Weimar, Germany; guido.morgenthal@uni-weimar.de; 2 Civil Engineering Department, Oregon Institute of Technology, 3201 Campus Drive, Klamath Falls, OR 97601, USA

**Keywords:** iPhone, LiDAR, time of flight, ToF, sensor characterization, vibration, modal parameter measurement, data fusion

## Abstract

Portable depth sensing using time-of-flight LiDAR principles is available on iPhone 13 Pro and similar Apple mobile devices. This study sought to characterize the LiDAR sensing system for measuring full-field vibrations to support modal analysis. A vibrating target was employed to identify the limits and quality of the sensor in terms of noise, frequency, and range, and the results were compared to a laser displacement transducer. In addition, properties such as phone-to-target distance and lighting conditions were investigated. It was determined that the optimal phone-to-target distance range is between 0.30 m and 2.00 m. Despite an indicated sampling frequency equal to the 60 Hz framerate of the RGB camera, the LiDAR depth map sampling rate is actually 15 Hz, limiting the utility of this sensor for vibration measurement and presenting challenges if the depth map time series is not downsampled to 15 Hz before further processing. Depth maps were processed with Stochastic Subspace Identification in a Monte Carlo manner for stochastic modal parameter identification of a flexible steel cantilever. Despite significant noise and distortion, the natural frequencies were identified with an average difference of 1.9% in comparison to the laser displacement transducer data, and high-resolution mode shapes including uncertainty ranges were obtained and compared to an analytical solution counterpart. Our findings indicate that mobile LiDAR measurements can be a powerful tool in modal identification if used in combination with prior knowledge of the structural system. The technology has significant potential for applications in structural health monitoring and diagnostics, particularly where non-contact vibration sensing is useful, such as in flexible scaled laboratory models or field scenarios where access to place physical sensors is challenging.

## 1. Introduction

Ubiquitous computing and powerful sensors available in consumer mobile devices have the potential to displace more expensive measurement, data acquisition, and postprocessing workflows for vibration-based structural health monitoring. Light detection and ranging (LiDAR), implemented in the iPhone 13 Pro [1] and similar Apple devices, provides a means of identifying the 3D position of objects in the environment. The sensor was added to the iPhone to improve low-light focusing for photography and video and for applications in augmented and virtual reality environments. While numerous authors have explored this sensor for instantaneous distance measurements [2,3,4,5] and 3D reconstruction of static objects [6,7,8,9,10], few have characterized the sensor for direct measurement of vibrations in deformable bodies. This paper addresses the gap in the existing literature concerning the capabilities and limitations of the Apple LiDAR sensing system for measuring time-varying deformations. An experiment using the sensor for modal parameter estimation is included.

Mobile devices have evolved to carry a significant number of on-board sensors with the ability to conduct data acquisition and postprocessing on the device itself [11,12,13]. Several of the earliest studies of vibration measurements with mobile devices focused on the MEMS accelerometer [14,15,16]. These studies demonstrated the accuracy of the accelerometer as well as the benefit of having it in a mobile platform with cellular connectivity. Other contact-based methods include traditional accelerometers, strain gages, LVDTs, inclinometers, and other devices that attach physically to the structure of interest and that may influence the structural response and require a constant power supply [17,18,19]. Non-contact full-field displacement measurements can be performed with established technologies; computer vision methods such as optical flow and feature tracking, high-speed photogrammetry, digital image correlation, and motion magnification all use 2D video-based measurement without depth sensing [20,21,22,23].

The use of ToF sensors to determine modal characteristics of a structure was demonstrated by Silva et al., who employed a Microsoft Kinect range camera to capture dynamic point clouds at a 30 Hz sampling rate to accurately identify the first three modes and frequencies for a suspended rectangular steel plate [24]. Benedetti et al. compared measurements from a MEMS accelerometer to vibrations measured with a Kinect v2 and GPS receiver with the intent of combining these lower-cost sensors into a robust system for structural monitoring [25]. An early characterization of the Kinect platform was presented by Smisek et al., demonstrating its utility in improving 3D reconstruction and accuracy comparable to multi-view stereo methods, although they did not consider the sampling rate or dynamic applications [26].

Studies of iPhone LiDAR for performing 3D reconstruction of static scenes have concluded that the sensor and various scanning apps can produce 3D models with an absolute accuracy of 1 cm for objects on the order of a few meters [6]. While drift can become significant when scanning areas on the order of 10 to 100 m, it can be mitigated with well-designed scanning paths as well as with gimbals or other physical stabilization devices [7]. Järvenpää [27] conducted a metrological characterization of the LiDAR sensing system in the Apple iPad Pro (2020) for static measurements, concluding that while the sensor is not optimal, it has potential for measurement purposes. While this appears to be the most rigorous assessment of the static characteristics of the sensor, it does not address dynamic applications or limitations. Following Järvenpää, we recognize that the LiDAR sensing system has benefits in terms of its portability, availability, and size, and that it can displace more expensive or cumbersome equipment. Future development of mobile LiDAR technology in consumer devices may address several of the current limitations, especially those reported here.

The rest of this paper is organised as follows. Section 2 describes the characteristics of the LiDAR sensing system shipped onboard the iPhone 13 Pro and discusses the geometrical properties, the sensing technology, and the processing steps involved in producing RGB-D depth maps. Section 3 provides details on the LiDAR sensing system’s characteristics in static and dynamic conditions and identifies optimal conditions for accurate measurement. In Section 4, the ideal measuring conditions are used as a baseline for experimental system identification of a 1.5-m long steel cantilever. Finally, Section 5 provides final remarks and discusses the necessary developments to improve the LiDAR sensing system’s reliability regarding its use for structural dynamics and modal identification.

## 2. iPhone 13 Pro LiDAR Properties

**Figure 1:**
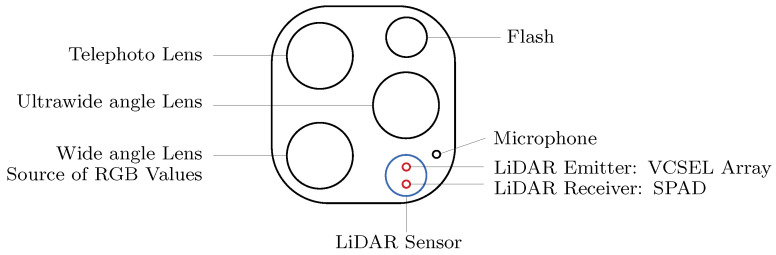
Arrangement of the iPhone 13 Pro camera cluster, including the location of the LiDAR emitter and receiver.

The rear-facing LiDAR sensor on the iPhone 13 Pro is in the lower right corner of the camera cluster, as shown in Figure 1. The sensor uses time-of-flight (ToF) principles and consists of a source of photons, or emitter, and a receiver. The emitter has 16 stacks of 4 vertical cavity surface emitting laser (VCSEL) cells, for 64 in total. The 64 laser pulses are multiplied by a 3 × 3 diffraction optical element (DOE) to make up 576 pulses [6]. The 576 laser pulses rebounded from object surfaces are detected and the individual time elapses are measured by a single-photon avalanche diode (SPAD) image sensor. Several methods for depth data processing in LiDAR systems are available in the literature [13,28,29]. The 576 depth points are then combined through a proprietary data fusion process with RGB values from the wide angle lens to produce a 256 × 192 depth map at 60 Hz [30,31]. Apple has released the access API to the 256 × 192 depth map, though not to the 576 depth points [32]. This depth map is accessed using StrayScanner, a freely available app, which is exclusively employed in this paper. Further details on StrayScanner are discussed in Section 3. Other apps are available to measure single values aggregated within a user-identified patch (e.g., PhyPhox) or create far denser dynamic point clouds that employ interpolation and smoothing algorithms (e.g., Record3D).

**Figure 2:**
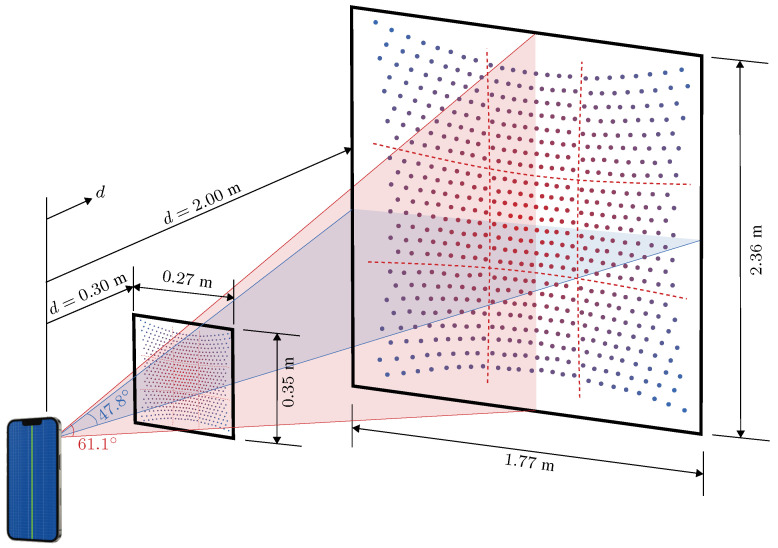
Distances and field of view required in order for objects to be reliably measured with the iPhone LiDAR sensing system. The 3×3 grid of 64 laser emitters each is defined by the dashed red lines.

A good graphical representation of the matrix of points emitted from the iPhone LiDAR, as captured by a near-infrared camera, is provided by Teppati et al. in [33]. Allain measured the camera field of view as being 71.9° by 53.9° [34], while Järvenpää identified the field of view of the sensor and camera as approximately 61.1° by 47.8° [27] while noting that the total illumination power of each individual laser pulse is dependent on the radial distance from the centre of the field of view. Based on the LiDAR sensing system characterization conducted here, the spatial bounds required in order for a vibrating object to be accurately measured using the iPhone LiDAR range from 0.30 m to 2.0 m distance. With a field of view for the wide RGB lense of 61.1° vertical and 47.8° horizontal, the maximum size of the object should fall within the bounds depicted in Figure 2.

## 3. Sensor Characterization

### 3.1. Problem Statement

The majority of the studies on LiDAR involve dedicated sensors specifically built for particular applications; these are generally quasi-static, i.e., there is no significant movement of the measured target during the LiDAR operation. The problems we address in this study are twofold:

(i) We characterise the LiDAR sensing system available on the iPhone 13 Pro and similar Apple devices regarding its static measurement properties, exploring its accuracy with different phone-to-target distances, noise floors, and lighting conditions.
(ii) We define the dynamic characteristics and capabilities of the sensor regarding dynamic accuracy, range, and sampling rate effects, and further relate these to applications and limitations of LiDAR in modal analysis.

To this end, in Section 3.2 the effects of different lighting conditions and measurement distances are investigated with respect to a static target (Objective i). Section 3.3 investigates several dynamic characteristics and provides guidelines on optimal measurement setup conditions and sensor limitations (Objective ii). LiDAR datasets throughout this study are collected with Stray Scanner, an iOS app available from Apple’s App Store that collects RGB-D data. Stray Scanner provides a depth map in a grid of 192×256 points along with RGB camera images, position estimates, and calibration matrices for each frame, and uses five sampling rate options: 1 Hz, 5 Hz, 15 Hz, 30 Hz, or 60 Hz. Unless otherwise specified, the datasets in this study have been collected in well-illuminated ambients, with the phone in a static condition and without user interference during measurement. In cases where LiDAR measurements are collected in a dynamic condition, such as when the operator holds the phone in their hand, the vibration signal may be obtained by computing the differential movement between the LiDAR data and the phone itself obtained via, e.g., the built-in accelerometer and gyroscope sensors [35,36,37].

### 3.2. Static Measurement Characteristics

**Figure 3:**
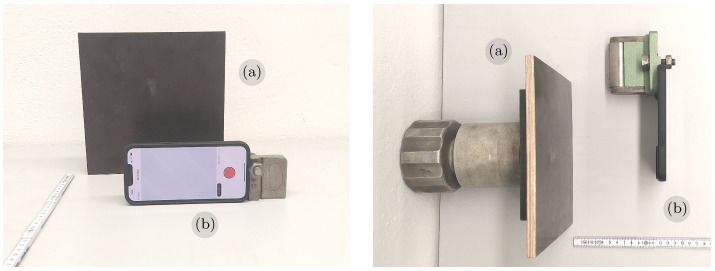
Experimental setup for static measurements, showing the rectangular dark-surface plate (**a**) used as a target for measuring the iPhone LiDAR (**b**). Front view (**left**) and top view (**right**).

First, we examine the static characteristics of the LiDAR sensing system of the iPhone 13 Pro. The experimental arrangement comprises a dark rectangular plate positioned with backside support, and the phone is affixed to a movable support at a distance *d* from the plate. Figure 3 illustrates the front and top perspectives of this setup.

**Figure 4:**
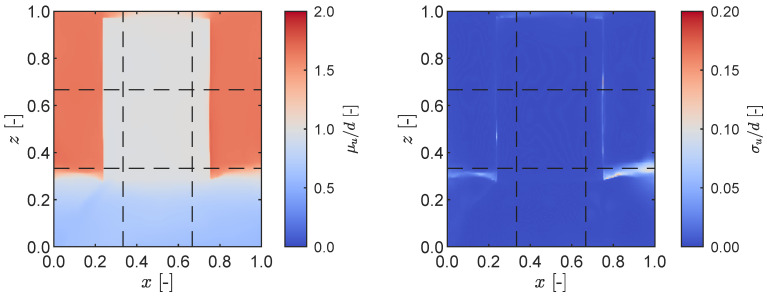
LiDAR measurement on a static plate with d=30 cm, showing the normalised mean displacement field (**left**) and its standard deviation (**right**). The dashed black lines indicate the LiDAR’s 3×3 grid divisions.

Each LiDAR reading comprised a depth map consisting of a grid measuring 192×256 points generated utilizing Apple’s ARKit API [38]. In all static tests, the measurement statistics were calculated based on a total of 300 individual readings. An illustration of the normalized mean and standard deviation of a LiDAR depth reading is presented in Figure 4, where the phone was positioned at a distance of d=30 cm from the target plate. Overall, a notable agreement was observed between the depth readings and the anticipated values, particularly in the mean sense; however, a bleeding effect occurred at the top of the frame due to the limited number of readings from the background, as the plate occupied almost the entire measured field of view. This effect was less pronounced on the sides of the target plate where more data was available. A distinct indication of a discontinuous boundary was observed, manifesting as a sharp increase in the measurement standard deviation. In contrast, along the bottom edge of the plate, where a continuous boundary existed between the plate itself and its support, the measurements remained stable and no increase in the standard deviation was observed.

**Figure 5:**
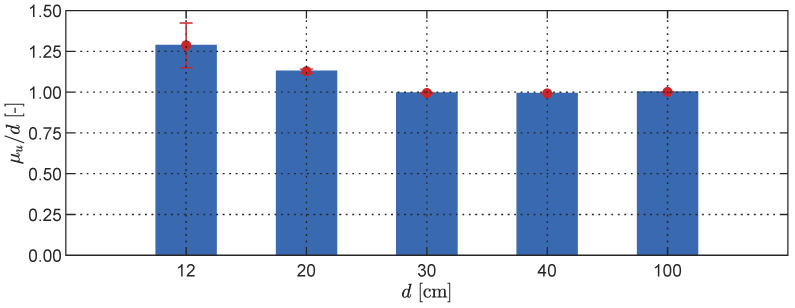
Histogram of normalised measurement mean and standard deviation (red error bars) for different phone-to-target distances *d*.

Considering that the LiDAR operates based on the time-of-flight (ToF) principle, it is to be expected that the measurement accuracy would vary with the distance between the sensor and its target. To investigate this effect, we conducted a static experiment with multiple phone-to-target distances *d* and compared the statistical properties of the measurements at the central point of the captured frames, i.e., x=y=0.50. The results of this analysis are shown in Figure 5, revealing a minimum separation distance of 30 cm, beyond which no significant decline in measurement quality was observed for distances up to 1.00 m. Furthermore, the signal-to-noise ratios (SNRs) were computed for all cases and were found to increase as the separation distance grew. The corresponding values are provided in Table 1.

**Figure 6:**
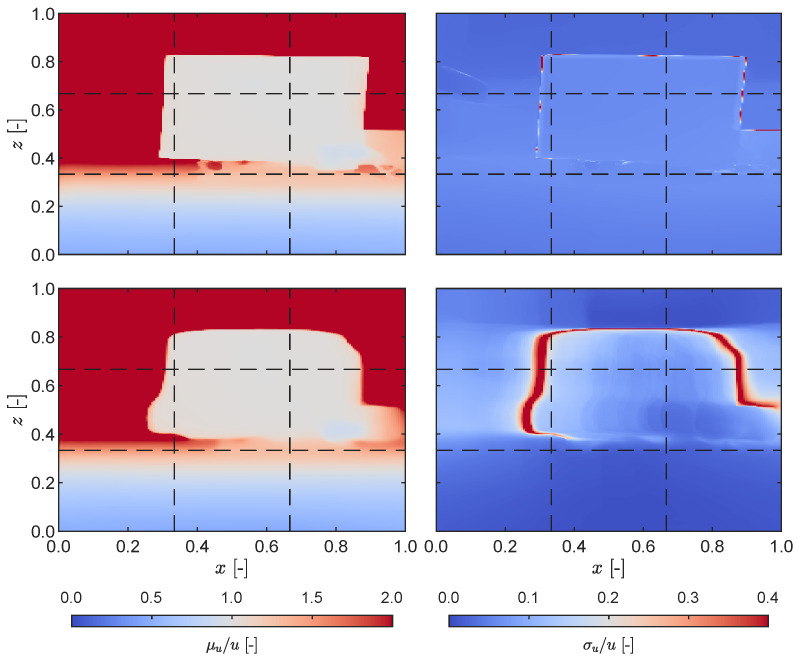
Static measurement: mean field (**left** column) and standard deviation (**right** column) for measurements with lights turned on (**top** row) and off (**bottom** row).

One notable characteristic of LiDAR sensors is their ability to perform well in dark or poorly illuminated environments, which is mostly related to the detection probability phenomenon in the SPAD [39,40]. In order to investigate this capability on the iPhone sensing system, we conducted measurements on the plate under two distinct lighting conditions, namely, well-illuminated and complete darkness. The statistical outcomes derived from the resulting depth maps are presented in Figure 6. Contrary to expectations, the depth map generated under normal ambient light exhibited greater accuracy in correctly identifying the rectangular shape of the plate. The standard deviation was observed to increase primarily at the boundaries between the plate and the background. In contrast, under dark conditions the plate’s shape was not accurately identified, and a wider area with high standard deviation values was observed. This effect arises from combining the RGB and depth data; during this process, the camera’s information is used to interpolate and colourize point clouds generated in combination with LiDAR depth measurements [33]. Similar findings were reported in [27], where instead of a dark environment the LiDAR depth information was recorded while the RGB camera was covered. Conversely, tests presented in [41] indicated that depth data could be obtained from RGB information only, with the LiDAR sensor covered, albeit with a reduced quality. This phenomenon can likely be attributed to Apple’s sensor fusion pipeline [30], which integrates raw data from multiple sensors before delivering depth information to the user. Apart from sensor fusion, other factors that may adversely affect LiDAR measurement data have been reported in literature including the target surface characteristics and geometry, sensor properties, and environmental effects [42].

### 3.3. Dynamic Properties

#### 3.3.1. Setup

In order to assess the dynamic characteristics of the LiDAR sensing system, we employed a linear air-bearing shaker (APS 113-AB) to mount the rigid plate mentioned in Section 3.2. Coupled with the Spektra VCS 400 vibration control system, this shaker is capable of imposing controlled motion on the system. It has a maximum payload capacity of 1.5 kg and can generate a peak-to-peak displacement range of 158 mm with a maximum oscillation frequency of 200 Hz. In addition, it can generate single-harmonic waves with specified root mean square (RMS) values or random motion within a specified spectrum.

**Figure 7:**
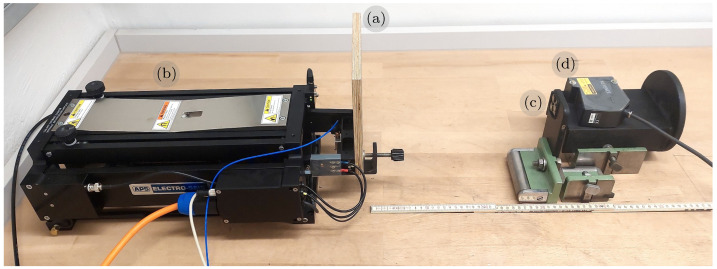
Experimental setup for dynamic measurements: the rectangular dark-surface plate (**a**) is mounted on a shaker (**b**) and used as a target for both LiDAR (**c**) and a laser displacement transducer (**d**).

The depth information collected with the iPhone’s LiDAR sensing system is internally combined with RGB data, and is obtained at a sampling frequency of 60 Hz [41]. Consequently, the maximum identifiable frequency is limited to 30 Hz, in accordance with the Nyquist–Shannon sampling theorem [43]. To compare and verify the processed results, we simultaneously utilized a laser displacement transducer (LDT) with a prescribed sampling rate of 200 Hz. A visual representation of the experimental setup is presented in Figure 7.

#### 3.3.2. Accuracy and Noise Characterisation

**Figure 8:**
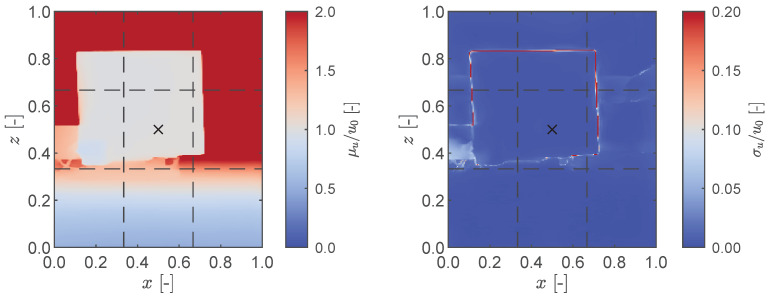
Depth map of LiDAR measurements of a rectangular plate under harmonic oscillation with f=2 Hz, showing the normalised mean displacement (**left**) and standard deviation (**right**). The black cross represents the middle of the LiDAR’s field of view.

**Figure 9:**
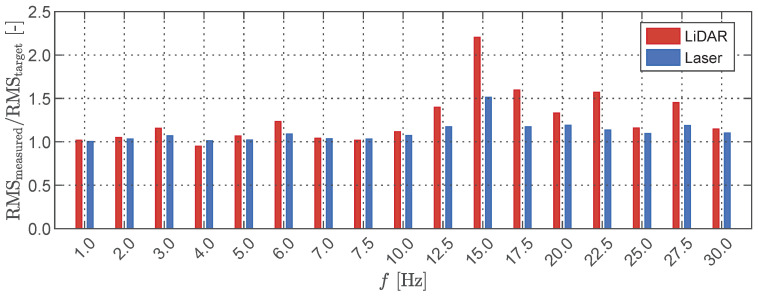
Normalised RMS of the LiDAR and laser sensors for a plate oscillating harmonically with different frequencies; LiDAR measurements were taken at the center of the field of view.

An initial investigation focused on assessing the accuracy and noise characteristics of the LiDAR sensing system when measuring dynamic targets. To this end, we subjected the target plate to a forced harmonic oscillation at various frequencies while simultaneously recording the motion using both the LiDAR sensor and the laser transducer. Both sensors were positioned at a distance of 35 cm from the target plate. After analyzing the normalized statistical properties of the LiDAR measurements, as illustrated in Figure 8, we reached similar conclusions to those obtained in the static case (cf. Figure 4). Notably, we observed an even sharper increase in the standard deviation along the edges of the plate. Using the centre of the LiDAR’s field of view as the reference point (indicated by the black cross in Figure 8), the root mean square (RMS) displacements from both the LiDAR sensing system and the laser displacement transducer were compared with the input RMS values specified in the shaker. The results are presented in Figure 9, indicating satisfactory agreement between the measurement data acquired from both sensors and the RMS values provided by the shaker for oscillation frequencies below 10 Hz. It is worth noting, however, that the LiDAR measurements exhibit a slightly greater deviation from the true RMS when compared to the laser displacement transducer. As the frequency exceeds 10 Hz, a significantly higher error is observed in the LiDAR measurements. This discrepancy is particularly evident at a frequency of 15 Hz, where the laser displacement transducer exhibits an increase of over 50% in RMS compared to the shaker input. This leads to the conclusion that the shaker itself introduces additional harmonic components at that specific excitation frequency.

**Figure 10:**
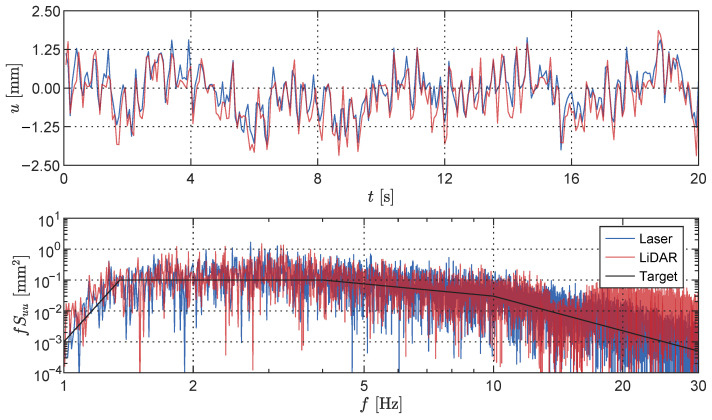
Broadband vibration of the rectangular plate, showing the measurement time history of the LiDAR and laser displacement transducer sensors (**top**) along with their respective frequency content and the target spectrum provided to the shaker (**bottom**).

Furthermore, a comparison between the LiDAR sensing system and the laser displacement transducer (LDT) was conducted for a broadband signal consisting of frequencies ranging from 1 Hz to 60 Hz. In this scenario, the shaker was provided with a target spectrum as the input and measurements were simultaneously obtained from both the LiDAR sensing system (at the centre of the field of view) and the LDT. The recorded time histories are presented in Figure 10 (top). Strong agreement is observed between the two signals; the depth measurements obtained from the LiDAR sensing system generally exhibit higher absolute amplitudes compared to the LDT measurements. This observation is further confirmed by the power spectral density (PSD) plot depicted in Figure 10 (bottom). While both sensors successfully capture the target PSD specified as the input to the shaker, the LiDAR dataset demonstrates an increased spectral amplitude in the lower frequency range, particularly around 1 Hz. At a frequency of 15 Hz, a sharp decrease in amplitude is observed in the LiDAR measurements. Above this frequency, however, the LiDAR data exhibit significantly higher spectral amplitudes compared to the LDT dataset, corroborating the higher RMS values illustrated in Figure 9. From further noise characterisation tests, it was observed that the spectral noise floor at the centre of the field of view measured approximately 5.3 mm/$\sqrt{\mathrm{Hz}}$ at a frequency of 1 Hz, with decreasing values for increasing frequencies.

#### 3.3.3. Phone-to-Target Distance

**Figure 11:**
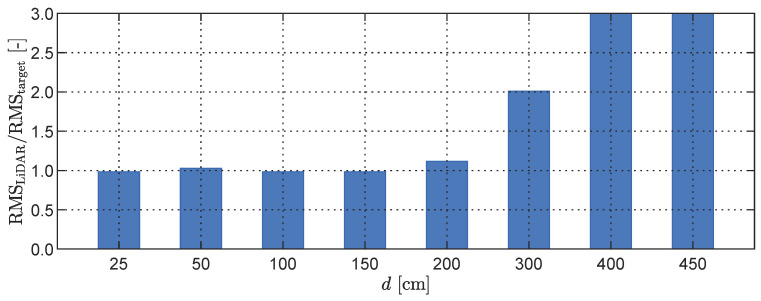
Measurement accuracy with respect to the normalised displacement RMS for varying phone-to-target distances *d*.

To further investigate the measurement accuracy of the LiDAR sensing system, we examined the impact of varying phone-to-target distances for a fixed oscillation frequency of 4 Hz, which falls within the reliable RMS identification range of the LiDAR. The results presented in Figure 11 compare the input RMS values provided by the shaker to the measurements obtained from the LiDAR.

For distances shorter than d=150 cm, the LiDAR sensing system can consistently and reliably identify the signal RMS, with an average error of less than 2%. However, when the separation distance is increased to 200 cm, the error rises significantly to 11%. At a distance of d=300 cm, the measured data become effectively unusable, with no reliable oscillation information discernible from the depth map. In cases where the phone-to-target distance exceeds 300 cm, the LiDAR sensing system struggles to accurately distinguish between the rectangular plate and the background within the field of view. Consequently, no meaningful oscillation information can be obtained from the depth map, rendering the data unreliable for analysis.

#### 3.3.4. Effective Sampling Rate

We proceeded to evaluate the LiDAR sensing system’s sampling rate properties. The sampling interval was examined using a Monte Carlo (MC) approach that encompassed eighteen individual measurements each lasting approximately 83 s, resulting in a total measurement duration of approximately 1500 s. The mean sampling rate obtained from these measurements is $\mu_{f_{s}}=59.9805$ Hz, with a standard deviation of $\sigma_{f_{s}}=0.0007$ Hz. Among all the sampled data, only 0.28% fell outside the 99% confidence interval of the MC analysis.

**Figure 12:**
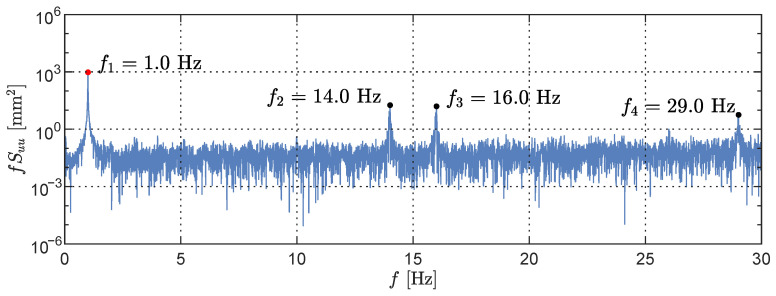
Power spectral density of LiDAR dataset of a forced oscillation test with $f_{1}=1.0$ Hz (red point). Aliases (black points) are observed at $f_{2}=14.0$ Hz, $f_{2}=16.0$ Hz, and $f_{3}=29.0$ Hz.

Subsequently, we investigated the frequency content of the forced oscillation scenario with an oscillating frequency of $f_{o}=1.0$ Hz, Following the Nyquist–Shannon sampling theorem [43], when considering the identified sampling rate the theoretical maximum frequency in the single-sided power spectral density (PSD) is 30 Hz. In light of the experimental conditions, we anticipated a single spectral peak at $f_{1}=1.0$ Hz. However, the PSD plot displayed in Figure 12 reveals three additional peaks at $f_{2}=14.0$ Hz, $f_{3}=16.0$ Hz, and $f_{4}=29.0$ Hz, indicating the presence of aliasing in the signal. Similar findings were observed for all other forced vibration oscillations cases, with the tested frequencies matching the ones presented in Figure 9).

**Figure 13:**
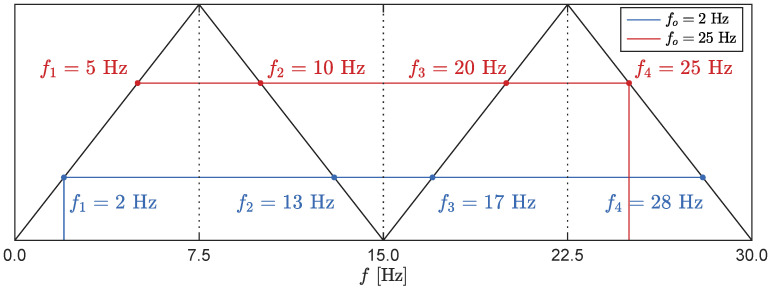
Schematic of the single-sided spectrum of a signal collected with $f_{s}=15$ Hz and upsampled to $f_{s}=60$ Hz without filtering. A true harmonic $f_{o}$ generates four spectral peaks at frequencies $f_{1}$ to $f_{4}$, and their values can be calculated according to the mirroring operations around 7.5 Hz and 15 Hz, depicted as black solid lines.

Upon further investigation, we hypothesized that the accurate internal LiDAR sampling rate would amount to $f_{s}=15$ Hz, with the collected data subsequently upsampled to match Apple’s RGB video camera sampling rate of 60 Hz. In such a case, the two-sided frequency spectrum would be mirrored at the Nyquist frequency, $f_{N}=7.5$ Hz, and any harmonic content above this frequency would appear as an alias below $f_{N}$. Additionally, upsampling the signal to $f_{s}=60$ Hz introduces two additional mirroring operations in the frequency spectrum, occurring at f=15 Hz and f=30 Hz. By following this operation, the true and alias peak locations for all forced oscillation frequencies $f_{o}$ can be calculated. A schematic of this procedure is depicted in Figure 13 for two forced oscillation cases, $f_{o}=2$ Hz and $f_{o}=25$ Hz.

**Figure 14:**
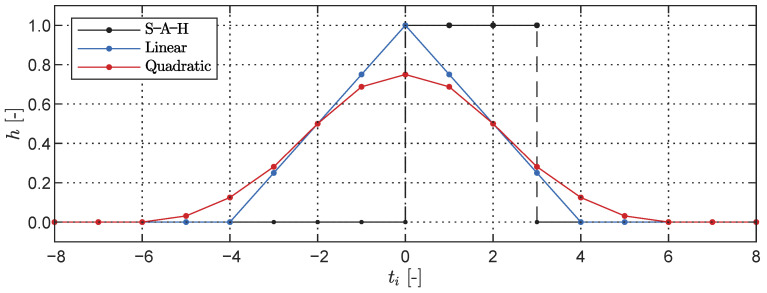
Interpolation kernels for convolution-based upsampling from $f_{s}=15$ Hz to $f_{s}=60$ Hz.

To validate the assumption of upsampling, synthetic single harmonic signals denoted as $u_{15}$ with amplitudes matching the experimental tests were created using a sampling frequency of $f_{s}=15$ Hz. Different interpolation techniques were then employed to upsample the analytical signals to a sampling frequency of $f_{s}=60$ Hz. The interpolation process was performed in the time domain through the following convolution operation:

$$u_{60}=h*u_{15},$$

where $u_{60}$ represents the upsampled signal at $f_{s}=60$ Hz, ∗ denotes the convolution operator, and *h* denotes the convolutional kernel that determines the type of interpolation. Three distinct strategies were tested: sample-and-hold (S-A-H), linear interpolation, and smoothed quadratic interpolation. The convolutional kernels *h* as functions of the time step $t_{i}$ are depicted in Figure 14.

**Figure 15:**
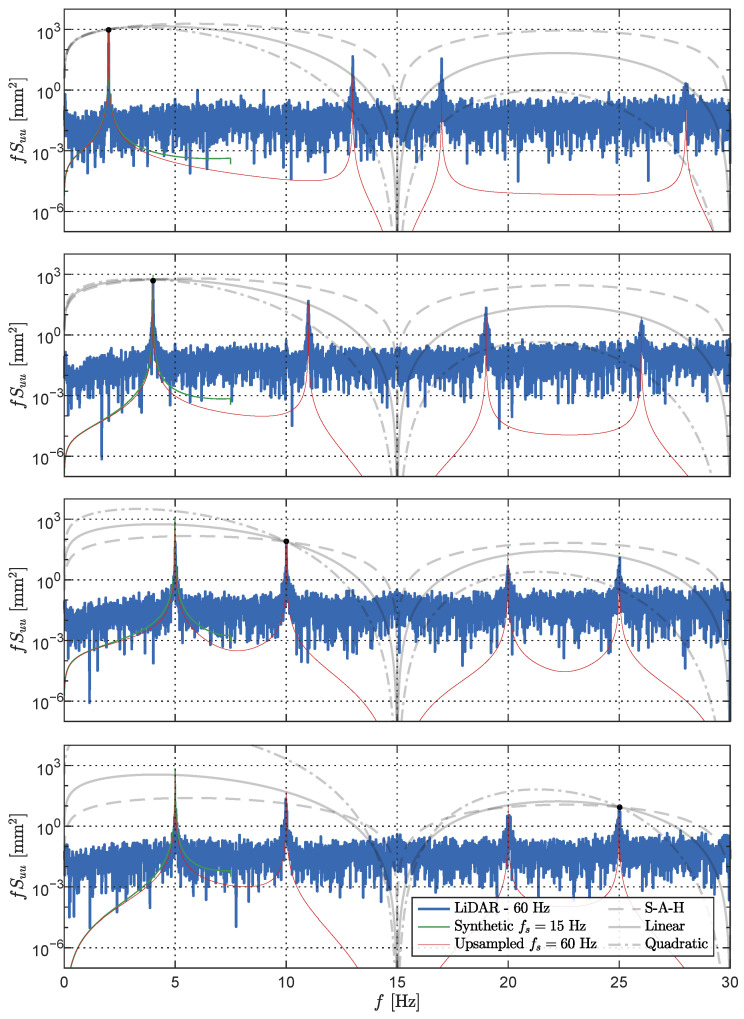
Convolution-based upsampling of single harmonic signals with $f_{o}=2.0$ Hz (**top**), $f_{o}=4.0$ Hz (**mid-top**), $f_{o}=10.0$ Hz (**mid-bottom**), and $f_{o}=25.0$ Hz (**bottom**). A comparison is shown between the LiDAR measurements, a synthetic signal with $f_{s}=15$ Hz, and its linear upsampled version at $f_{s}=60$ Hz. The frequency response of the three interpolation kernels is shown for comparison.

For further analysis, we selected the upsampling results corresponding to forced oscillation frequencies of $f_{o}=2.0$ Hz, $f_{o}=4.0$ Hz, $f_{o}=10.0$ Hz, and $f_{o}=25.0$ Hz, as depicted in Figure 15. Initially, we verify the assumption of a true sampling frequency of $f_{s}=15$ Hz. The expected spectral peaks after upsampling were calculated with the procedure shown in Figure 13, and the resulting frequencies $f_{1}$ to $f_{4}$ are presented in Table 2. Comparing the calculated peak frequencies with the experimental results displayed in Figure 15 confirms the upsampling assumption and provides a clear explanation for the observed aliases in the experimental dataset.

In addition to assessing the presence of aliases in the upsampled signal, the convolutional kernels *h* can aid in explaining the magnitude of the peaks observed in the signal’s power spectral density (PSD). Their frequency domain representation can be interpreted as a linear transfer function between the original signal and the upsampled signal, providing insights into the peak amplitudes. Subsequently, we compared the frequency response of each convolutional kernel to the spectral amplitudes of the expected frequency $f_{1}$ and the aliases $f_{2}$, $f_{3}$, and $f_{4}$. The findings presented in Figure 15 reveal a relationship between the interpolation order and the frequency content, potentially influenced by Apple’s sensor fusion pipeline [30]. While the sample-and-hold interpolation method yields higher frequency amplitudes, it fails to fully explain the measured data. Notably, for the case of $f_{o}=2.0$ Hz the linear interpolation kernel results in lower spectral amplitudes at the aliases, with the peaks lying between the linear and S-A-H methods. Conversely, for $f_{o}=25$ Hz the spectral amplitudes are best accounted for by the quadratic interpolation procedure. In general, between the three interpolation options, the linear kernel explains the data best and closely matches the spectral content observed in the LiDAR measurements across a broad range of frequencies.

In practical measurements, there is no straightforward method to revert the upsampling process and accurately distinguish between the true frequencies and the aliases. The current LiDAR measurements are most effective for signals with frequency content limited to 7.5 Hz, which can be accurately identified by applying low-pass filtering and downsampling to the signals collected at $f_{s}=60$ Hz. For signals with higher frequency content, however, prior knowledge about the underlying process that generated the measurements is essential to accurately discern the true harmonics from the aliases. This is demonstrated by the experiment described in the next section.

## 4. Experiment

### 4.1. Setup

**Figure 16:**
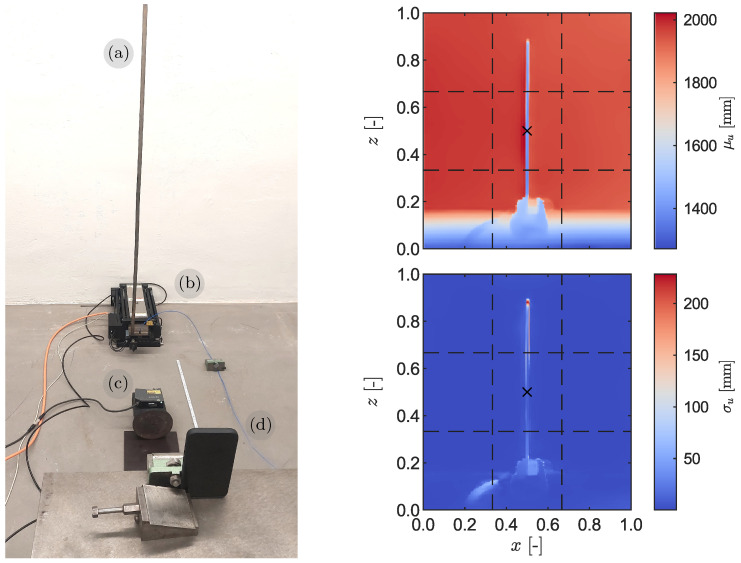
(**Left**): the steel cantilever (**a**) mounted on the shaker (**b**) with the laser displacement transducer (**c**) at the shaker level and the iPhone 13 Pro (**d**) 1.50 m away from the cantilever at a height approximately equal to its centre. (**Right**): the mean (**top**) and standard deviation (**bottom**) of a LiDAR depth map measurements. The black cross represents the middle of the field of view.

We now present an application of the iPhone’s LiDAR sensing system for the modal identification of a steel cantilever structure with a 16 mm by 2 mm cross-section and a height of H=1.50 m. The cantilever was mounted on the shaker and subjected to a forced random base oscillation. To monitor the displacement at the base of the cantilever, the laser displacement transducer was mounted at the shaker lever. Simultaneously, the iPhone was mounted at a height corresponding to the centre of the cantilever and positioned at a distance of d=1.50 m away from it. A schematic illustration of the experimental setup and a sample of the LiDAR’s depth map displaying both the mean and standard deviation are depicted in Figure 16.

**Figure 17:**
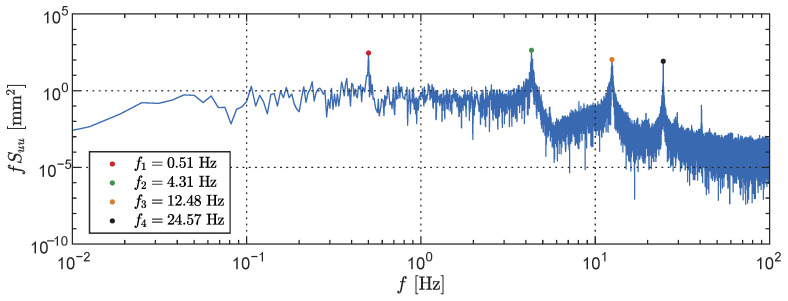
Power spectral density of a cantilever white noise response produced with measurements from the laser displacement transducer at a height of 1.00 m. The first four natural frequencies are used as a benchmark for further comparisons.

**Figure 18:**
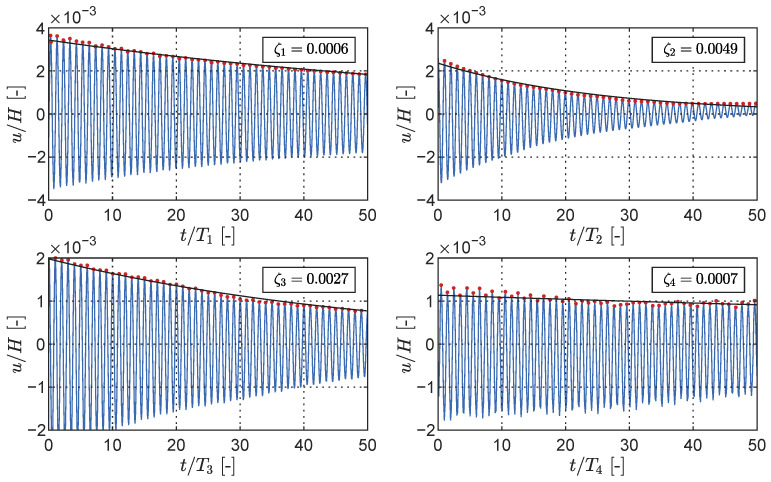
Modal damping ratio identified via logarithmic decrement for the four first cantilever modes using the laser transducer measurements (for the frequencies, see Figure 17). The time *t* is normalised with the modal period $T_{i}=1/f_{i}$, while the displacement *u* is normalised with the cantilever height *H*.

The modal properties of the cantilever were initially determined using the LDT only. By positioning the laser transducer at a height of 1.00 m and conducting a random oscillation test, the first four natural frequencies were identified based on the response power spectral density (PSD), as illustrated in Figure 17. Additionally, forced oscillation simulations were performed at these four identified frequencies. When the shaker was stopped, the free decay oscillation was recorded to determine the damping ratios through logarithmic decay, as displayed in Figure 18. The natural frequencies and damping ratios obtained from the LDT tests serve as a foundation for comparison with the results derived from the LiDAR depth map.

### 4.2. Data Preprocessing

The collected depth map covers the entire LiDAR field of view, as depicted in Figure 16. Because the cantilever can be regarded as a line-like structure, the initial preprocessing step involves identifying an optimal coordinate in the horizontal (*x*) direction that encompasses readings across the height of the cantilever. Subsequently, the top-most point of the cantilever was determined as the first *z* coordinate containing readings compatible with the static distance between the cantilever and the iPhone, which is approximately 1.50 m. The base point was determined by comparing the cross-correlation coefficients between several candidate LiDAR points in the field of view and the measurements obtained from the laser displacement transducer (LDT). When the boundaries were identified, a total of 171 out of the 256 depth measurement points remained in the dataset, which were uniformly distributed across the height of the cantilever. To centre the depth measurements along a vertical line, the mean value was subtracted from each datapoint.

**Figure 19:**
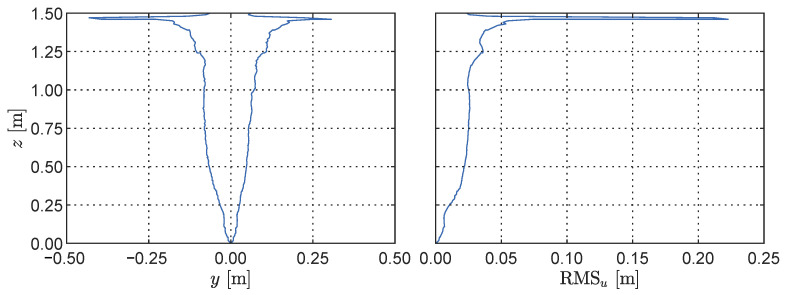
Statistics of the LiDAR depth measurements for the cantilever case: envelope of the displacements across the cantilever height (**left**) and the corresponding root mean squares (**right**).

**Figure 20:**
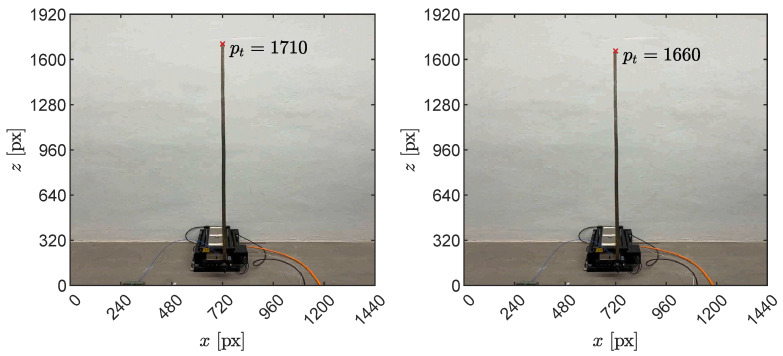
Snapshots of the cantilever during dynamic base oscillation with its tip on high (**left**) and low (**right**) heights according to the instantaneous dynamic configuration. The corresponding pixel $p_{t}$ is marked for reference.

The processed depth map statistics depicting the envelope and root mean square (RMS) of readings across the cantilever height are displayed in Figure 19. As expected for the structural model, a general increase is observed in both the RMS and the response envelope as the height increases. Close to the top, however, a sharp increase is observed, with a maximum absolute amplitude of over 40 cm and an RMS of 22 cm. This phenomenon occurs due to the tip of the cantilever periodically entering and exiting the field of view, depending on its dynamic response to the base oscillation. Figure 20 shows two snapshots recorded during a dynamic simulation, with a reference point marking the top pixel to showcase the effect. To mitigate the impact of this effect, the topmost part of the depth map is further clipped during preprocessing to remove the unreliable portion of the data.

**Figure 21:**
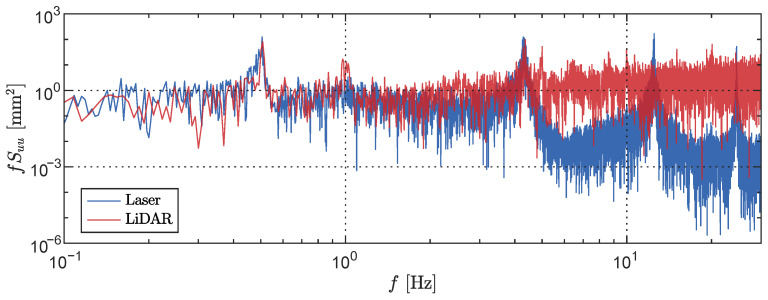
Power spectral density comparison between the laser displacement transducer and the LiDAR depth measurements collected at a height of z=1.00 m.

Next, we proceeded to compare the power spectral density of the laser displacement transducer measurements at a height of z=1.00 m with those obtained from the LiDAR sensing system. The comparative results are illustrated in Figure 21. The PSD peaks corresponding to the first two cantilever natural frequencies from the LiDAR data are in agreement with the PSD values derived from the LDT data. However, the LiDAR data exhibit an additional peak near 1 Hz, as well as several others at higher frequencies. However, the LiDAR measurements fail to match the two highest natural frequencies of the cantilever, which exceed the true LiDAR Nyquist frequency of $f_{N}=7.5$ Hz. Moreover, the PSD results indicate a significantly higher RMS for the LiDAR measurements compared to the LDT data, suggesting a higher level of noise in the former. This increased noise level may be a contributing factor to the LiDAR’s inability to accurately capture the higher frequencies of the cantilever structure.

### 4.3. Modal Analysis

After completing the preprocessing steps and eliminating the bottom and tip cantilever parts, the depth dataset contained 119 of the initial 171 measurement points in a regular grid spanning from H=18 cm to H=140 cm. The spatial resolution was determined by the distance between the iPhone and the cantilever; refer to Figure 2 for details. The data were originally collected at a sampling frequency of $f_{s}=60$ Hz and subsequently downsampled to $f_{s}=15$ Hz to correspond to the true sampling rate of the LiDAR system.

Modal analysis was conducted using the Stochastic Subspace Identification (SSI) method [44,45]. Instead of employing the complete depth dataset for SSI, we opted for a Monte Carlo analysis with 1000 iterations. In this approach, five out of the 119 possible heights were selected using a Latin Hypercube sampling approach [46]. Modal analysis was then individually performed for each of these selected heights. The rationale behind this procedure was to mitigate the influence of the substantial noise present in the depth dataset, which could potentially impact the correlation of close measurement points [47]. Furthermore, this methodology allows for a probabilistic perspective on the modal parameters, enabling the empirical estimation of statistical properties related to modal frequencies, damping ratios, and mode shapes.

**Figure 22:**
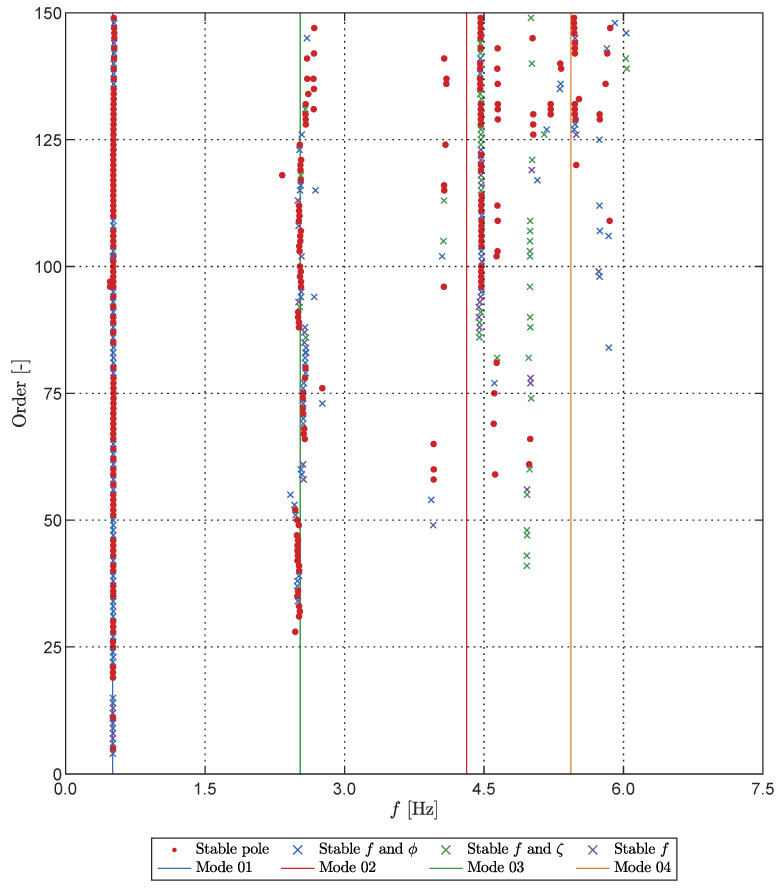
Stabilisation diagram for modal identification of the cantilever structure. Poles are classified according to their stability regarding the frequencies *f*, damping ratios ζ, and mode shapes ϕ. The expected pole locations (or aliases) are shown for each expected cantilever mode.

The stabilization diagram for one of the Monte Carlo cases is illustrated in Figure 22. The pole classification is based on the stability, wherein we examine the consistency of the modal frequencies *f*, damping ratios ζ, and mode shapes ϕ across different system orders while allowing for tolerable errors of $\epsilon_{f}=1$%, $\epsilon_{\zeta }=5$%, and $\epsilon_{\phi }=5$%. Additionally, Figure 22 displays the expected frequency location of each pole derived from the frequencies obtained through LDT analysis (cf. Figure 17). For natural frequencies exceeding the Nyquist frequency $f_{N}=7.5$ Hz, the expected pole frequency was computed using the procedure defined in Figure 13, with the pole selection based on the expected alias instead of the true structural frequency.

The results of the modal analysis in Table 3 reveal a satisfactory agreement between the frequencies identified from the LDT data and those derived from the LiDAR depth maps, particularly in terms of the mean values. The maximum difference observed in the second mode amounts to 0.14 Hz (3.2%). The standard deviation of the obtained frequencies increases with the mode number, which is attributed to the fact that the two highest frequencies appear as aliases in the frequency spectrum. Conversely, the damping ratio values obtained from the SSI analysis differ significantly from those obtained using the free vibration measurement data collected by the LDT, although both are small in magnitude. Such discrepancies are expected due to the presence of high noise content in the LiDAR signal, which generally affects the quality of modal identification, especially regarding damping ratios [48,49]. Furthermore, the core assumption of the SSI model is a general white noise excitation, which is violated in our experiment due to its consisting of a white noise base excitation. The unreliability of the identified damping ratios is further evident from the values of the corresponding standard deviations. For more accurate results, substantial improvements in LiDAR hardware performance are required in order to obtain data of higher quality.

**Figure 23:**
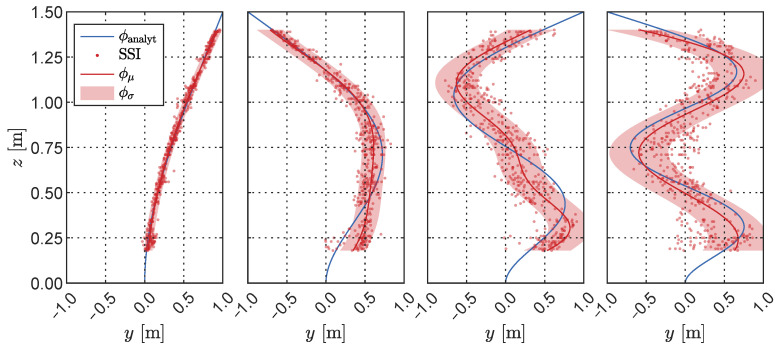
First to fourth (left to right) mode shapes obtained from the Monte Carlo SSI modal analysis. The Gaussian process regression fitting yielded a mean $\phi_{\mu }$ and standard deviation $\phi_{\sigma }$ for each mode shape, allowing for statistical analysis of the results. The cantilever analytical mode shapes $\phi_{\mathrm{analyt}}$ are shown for comparison.

In each of the 1000 Monte Carlo iterations, the mode shapes for all four modes were estimated at the five locations defined by each specific sample from the Latin Hypercube. Figure 23 displays the combined results obtained through SSI along with the analytical estimation of the cantilever mode shapes ϕ, calculated as follows:

$$\phi_{i}\left(z\right)=\sin\lambda_{i}z-\sinh\lambda_{i}z+\left(\cosh\lambda_{i}z-\cos\lambda_{i}z\right)\frac{\sinh\lambda_{i}H+\sin\lambda_{i}H}{\cosh\lambda_{i}H+\cos\lambda_{i}H}$$

where *z* is the height coordinate, *H* is the cantilever total height, and $\lambda_{i}$ is a coefficient for mode *i* obtained as the *i*-th solution of the equation $\cos\lambda_{i}H=-1/\cosh\lambda_{i}H$. To quantify the uncertainty associated with the mode shapes, we employed a Gaussian process (GP) [50] for fitting to the SSI results. The outcomes are expressed in terms of the GP’s mean and standard deviation, and are depicted in Figure 23.

**Figure 24:**
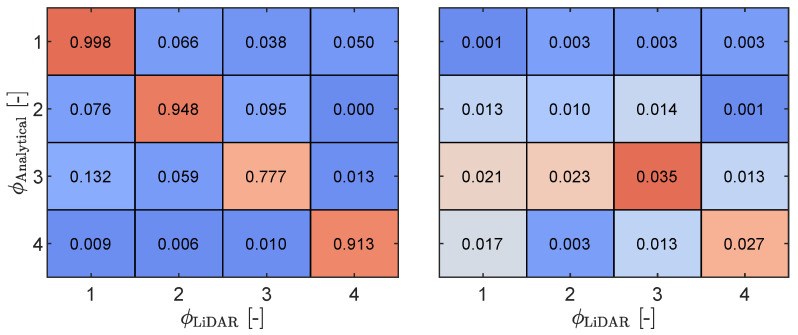
Modal assurance criterion mean (**left**) and standard deviation (**right**) based on samples derived from the Gaussian process fitting of the mode shapes. High to low values are indicated by the red to blue colors, respectively.

Strong agreement is observed between the analytical mode shapes and those identified by the SSI, particularly for the first, second, and fourth modes. By employing GP fitting, we quantitatively assessed the uncertainty associated with each mode based on the optimal identification of noise while considering the scattered modal points identified through SSI. Among the four modes, mode 1 exhibits the smallest noise standard deviation, amounting to $\phi_{\sigma ,1}=0.069$ m. Conversely, mode 2 is characterized by higher scattering, leading to a standard deviation of $\phi_{\sigma ,2}=0.225$ m. Mode 3 deviates more from its analytical counterpart in terms of the mean and has a higher standard deviation of $\phi_{\sigma ,3}=0.274$ m, while mode 4, despite a better mean result, exhibits a higher standard deviation of $\phi_{\sigma ,4}=0.326$ m. To further measure the similarity between the analytical and identified modes in light of their uncertainties, we utilized the modal assurance criterion (MAC). The results presented in Figure 24 were computed empirically by sampling from the Gaussian process fitting, as depicted in Figure 23. These results reaffirm the previously discussed uncertainty measurements, with mode 3 exhibiting a low MAC value when compared to its analytical counterpart. Moreover, modes 3 and 4 display higher levels of uncertainty. The increasing standard deviation with respect to the mode number again reflects the influence of the high noise content in the signal and the modal identification based on aliases, which significantly impacts the quality of modal identification.

## 5. Conclusions

In this paper, we have investigated the characteristics of Apple’s iPhone 13 Pro LiDAR sensing system while focusing on its optimal measurement distance, noise properties, and sampling rate properties. Additionally, an application of the sensor to the modal identification of a steel cantilever structure is presented.

Previous studies have highlighted the utility of LiDAR technology for static measurements. Our results indicate that a minimal phone-to-target distance of 30 cm yields optimal measurement values and an acceptable signal-to-noise ratio in static conditions. However, a counterintuitive finding was that accuracy decreases and uncertainty increases in poorly illuminated conditions. This is likely due to Apple’s internal combination of RGB data and depth information to generate a dense depth map, which adversely affects LiDAR readings under dark conditions. In practical applications, various additional external factors can potentially impact the quality of the acquired depth maps, including environmental conditions such as rain and fog. To enable the deployment of such a system, further investigation of these topics is necessary and constitutes a crucial area for future research.

For dynamic measurements, good displacement RMS values were obtained for phone-to-target distances up to 200 cm, indicating an optimal LiDAR measurement range between 30 and 200 cm. Nevertheless, even within this range the noise content in LiDAR measurements is significantly higher than in dedicated displacement sensors such as laser displacement transducers. An important outcome is that while LiDAR data are commonly output at a sampling frequency of 60 Hz to match the RGB camera, these data in fact have a sampling rate of 15 Hz. Upsampling is performed to match the target sampling frequency, introducing aliases to the frequency content of the depth measurements. Additionally, we observed that while the upsampling procedure is frequency-dependent, it can be approximated effectively via simple linear interpolation.

To demonstrate the LiDAR sensing system’s properties, an experiment involving the modal identification of a steel cantilever was conducted using covariance-driven Stochastic Subspace Identification in a Monte Carlo setting while providing confidence intervals for all modal properties. The identified natural frequencies matched those obtained from the laser displacement transducer readings with an average error of 1.9%, while the damping ratios significantly differed from those obtained from logarithmic decrement analysis, likely due to the high level of noise in the depth measurements. The mode shapes were further processed using a Gaussian process fitting, providing continuous averages and uncertainty ranges. After comparing them with an analytical counterpart model of the cantilever, high Modal Assurance Criterion (MAC) values were observed for the first two mode shapes, which lie below the Nyquist frequency of the LiDAR measurements. The two higher modes were identified as aliases in the true frequency content, exhibiting lower MAC values and higher standard deviations, again likely due to noise.

The investigated sensor holds potential applications in the fields of Structural Diagnostics and Structural Health Monitoring of civil and mechanical systems. In its current state, it enables efficient and portable noncontact displacement measurements, overcoming many logistical and operational issues associated with more traditional sensing strategies. In the context of civil engineering applications, the present limitations of phone-to-target distance and field-of-view dimensions confine the practical utility of this sensor technology to flexible structural components, where the analysis of frequency content is of primary importance. Such components include structural cables in suspension and cable-stayed bridges as well as external post-tensioning tendons. The LiDAR sensing system can find a diverse range of applications in laboratory settings, particularly in the study of slender scaled structural models. The noncontact property is of particular advantage in such contexts, as it avoids any potential alterations to the structural dynamic properties. Enhancements to the sensor, such as increased phone-to-target distances and improved signal-to-noise properties, along with the provision of raw depth data before RGB interpolation, could further enhance its usefulness for monitoring purposes.

## Figures and Tables

**Table 1:** Measurement properties for static depth maps at different sensor-to-target distances.

Target Distance	Measurement		
[cm]	$u_{\mu }$ [cm]	$u_{\sigma }$ [cm]	SNR [dB]
12	15.4	0.65	27.6
20	22.6	0.10	46.7
30	29.9	0.05	55.5
40	39.7	0.03	63.0
100	100.1	0.09	61.4

**Table 2:** Forced oscillation frequencies $f_{o}$ with the expected frequency content $f_{1}$ considering $f_{s}=15$ Hz. The aliases $f_{2}$, $f_{3}$, and $f_{4}$ are provided for further comparison with measured data.

$f_{o}$ [Hz]	$f_{1}$ [Hz]	$f_{2}$ [Hz]	$f_{3}$ [Hz]	$f_{4}$ [Hz]
2.0	2.0	13.0	17.0	28.0
4.0	4.0	11.0	19.0	26.0
10.0	5.0	10.0	20.0	25.0
25.0	5.0	10.0	20.0	25.0

**Table 3:** Modal analysis results for the cantilever structure. The means μ and standard deviations σ of natural frequencies *f* and modal damping ratios ζ identified from the Monte Carlo SSI procedure are compared to the laser displacement transducer (LDT) results. For natural frequencies higher than $f_{N}=7.5$ Hz, the identified frequency corresponds to the alias appearing below the Nyquist frequency.

Mode	Frequencies				Damping Ratios		
	$f_{\mathrm{LDT}}$	$f_{\mathrm{alias}}$	$f_{\mu ,\mathrm{LiDAR}}$	$f_{\sigma ,\mathrm{LiDAR}}$	$\zeta_{\mathrm{LDT}}$	$\zeta_{\mu ,\mathrm{LiDAR}}$	$\zeta_{\sigma ,\mathrm{LiDAR}}$
[-]	[Hz]	[Hz]	[Hz]	[Hz]	[-]	[-]	[-]
1	0.51	-	0.50	0.04	0.0006	0.0048	0.0026
2	4.31	-	4.45	0.08	0.0049	0.0139	0.0091
3	12.48	2.52	2.55	0.14	0.0027	0.0182	0.0099
4	24.57	5.43	5.48	0.27	0.0007	0.0012	0.0008

## Data Availability

The data presented in this study are openly available on Figshare at http://www.doi.org/10.6084/m9.figshare.23770569 (accessed on 8 September 2023).
